# Resting state functional connectivity predicts neurofeedback response

**DOI:** 10.3389/fnbeh.2014.00338

**Published:** 2014-09-24

**Authors:** Dustin Scheinost, Teodora Stoica, Suzanne Wasylink, Patricia Gruner, John Saksa, Christopher Pittenger, Michelle Hampson

**Affiliations:** ^1^Magnetic Resonance Research Center (MRRC), Department of Diagnostic Radiology, Yale School of MedicineNew Haven, CT, USA; ^2^Department of Psychiatry, Yale School of MedicineNew Haven, CT, USA; ^3^Department of Psychology, Yale UniversityNew Haven, CT, USA; ^4^Child Study Center, Yale School of MedicineNew Haven, CT, USA

**Keywords:** neurofeedback, real-time fMRI, resting state connectivity, obsessive-compulsive disorder, orbitofrontal cortex

## Abstract

Tailoring treatments to the specific needs and biology of individual patients—personalized medicine—requires delineation of reliable predictors of response. Unfortunately, these have been slow to emerge, especially in neuropsychiatric disorders. We have recently described a real-time functional magnetic resonance imaging (rt-fMRI) neurofeedback protocol that can reduce contamination-related anxiety, a prominent symptom of many cases of obsessive-compulsive disorder (OCD). Individual response to this intervention is variable. Here we used patterns of brain functional connectivity, as measured by baseline resting-state fMRI (rs-fMRI), to predict improvements in contamination anxiety after neurofeedback training. Activity of a region of the orbitofrontal cortex (OFC) and anterior prefrontal cortex, Brodmann area (BA) 10, associated with contamination anxiety in each subject was measured in real time and presented as a neurofeedback signal, permitting subjects to learn to modulate this target brain region. We have previously reported both enhanced OFC/BA 10 control and improved anxiety in a group of subclinically anxious subjects after neurofeedback. Five individuals with contamination-related OCD who underwent the same protocol also showed improved clinical symptomatology. In both groups, these behavioral improvements were strongly correlated with baseline whole-brain connectivity in the OFC/BA 10, computed from rs-fMRI collected several days prior to neurofeedback training. These pilot data suggest that rs-fMRI can be used to identify individuals likely to benefit from rt-fMRI neurofeedback training to control contamination anxiety.

## Introduction

Dysregulation of anxiety is a core component of many neuropsychiatric conditions. Obsessive-compulsive disorder (OCD) is characterized by intrusive obsessions, which are often associated with anxiety, and with repetitive compulsions that seek to control that anxiety (Jenike, 2004). One common presentation of OCD is characterized by extreme contamination anxiety, often triggered by thoughts or images of, or contact with, potential contaminates such as dirt, body secretions, or mold (Bloch et al., 2008). Improving control of contamination anxiety is a key step in improving the quality of life for many individuals with OCD.

Treatments for contamination anxiety and for OCD exist, but none are universally effective (Franklin and Foa, 2011). For individuals who do not respond to standard behavioral and pharmacological treatments, interventions that more directly modulate the specific brain regions whose dysfunction is implicated in the disorder may be of benefit. In extreme cases, this modulation is sometimes done through invasive procedures such as deep brain stimulation (Greenberg et al., 2010). Targeted brain modulation using neurofeedback via real-time functional magnetic resonance (rt-fMRI) may prove to be an alternative (Scheinost et al., 2013).

rt-fMRI neurofeedback involves monitoring the blood oxygenation level dependent (BOLD) signal, a measure of brain activity, and providing immediate feedback to the subject showing them how specific brain activity patterns are changing over time. This form of feedback can facilitate learned control over brain activity and associated behaviors (Sulzer et al., 2013; Ruiz et al., 2014). Neurofeedback training has shown promise as a potential treatment in several clinical disorders including addiction (Hanlon et al., 2013; Li et al., 2013), tinnitus (Haller et al., 2010), stroke (Sitaram et al., 2012), depression (Linden et al., 2012; Young et al., 2014), Parkinson’s Disease (Subramanian et al., 2011), and schizophrenia (Ruiz et al., 2013a,b). rt-fMRI neurofeedback can produce changes in brain function (Hampson et al., 2011; Harmelech et al., 2013) and related behaviors (Shibata et al., 2011). In individuals with significant but subclinical contamination anxiety, we have shown that neurofeedback of activity in the orbitofrontal cortex (OFC) and anterior prefrontal cortex Brodmann area (BA) 10 can reorganize functional brain networks associated with anxiety and reduce the anxiety produced by contamination-related stimuli (Scheinost et al., 2013).

Clinically, a trial of an intervention that ultimately proves ineffective carries substantial cost, in both time, resources, and ongoing patient suffering. How best to match an individual to an intervention is therefore a crucially important question. Predictors of response that can help with treatment selection in neuropsychiatric conditions such as OCD would be of enormous clinical value but have been slow to emerge.

Here, we ask whether resting-state fMRI (rs-fMRI) can predict response to neurofeedback training and, thus, potentially guide treatment selection in the future. Previous research suggests that imaging-based biomarkers can be used to predict performance with a brain-computer interface (Halder et al., 2013). rs-fMRI, in particular, provides a great opportunity for identifying biomarkers to aid clinical decisions, given that it can be collected in clinical populations without requiring any task performance and yet provides a wealth of information about brain function (Constable et al., 2013; Lee et al., 2013). To investigate whether brain connectivity at rest can predict reduction in contamination anxiety induced during a neurofeedback intervention, we correlated a voxel-wise measure of functional connectivity, computed from rs-fMRI collected prior to neurofeedback training, with behavioral response to the neurofeedback intervention in a cohort of healthy subjects with subclinical contamination anxiety (Scheinost et al., 2013). We then examined whether a similar relationship existed in a small cohort of patients.

## Methods

Data were from studies performed at Yale University School of Medicine, New Haven, CT. All protocols were reviewed and approved by Human Research Protection Program at Yale University. Written informed consent was obtained. All scans were obtained and analyzed at Yale University.

### Subjects

Two cohorts of subjects were used in this study. The first cohort has been described previously (Scheinost et al., 2013) and consisted of 10 subjects without any clinical diagnosis of OCD, but with high levels of contamination anxiety. Only the 10 subjects who received true neurofeedback in our previous study—not the 10 who received sham neurofeedback in the control condition—are included in the present analysis. The second cohort consisted of five OCD patients with moderate symptom severity (Table 1) and prominent contamination obsessions.

**Table 1:** **Clinical characteristics and symptom improvement in five OCD patients who underwent rt-fMRI biofeedback**.

	Subject					Average
	**1**	**2**	**3**^†^	**4**^†^	**5**^†^
**Sex**	F	M	F	M	M	3M/2F
**Age**	33	41	65	43	46	46
**Handedness**	R	L	R	R	R	4R/1L
**Other dx**	None	MDD	Past MDD	None	MDD
		Panic D/O			BDD
		GAD			Motor tic
		Past SUD			
**Psychiatric medications**	None	None	fluoxetine	None	None
			Synthroid			
			Immitrex*			
**Y-BOCS**
**Baseline**	27	28	25	26	28	26.8
**Midpoint**	24	21	23	25	23	23.2
**Final**	–	–	19	23	20	20.6
**Improvement**	11%	25%	24%	11.5%	28.5%	20%

MDD—major depressive disorder. Panic D/O—panic disorder, with agoraphobia. GAD—generalized anxiety disorder. SUD—substance use disorder (in remission). BDD—body dysmorphic disorder. * taken occasionally, as needed. ^†^ rs-fMRI data collected prior to neurofeedback and used in connectivity analysis; see Figure 2.

### Neurofeedback training

Healthy subjects and OCD patients received neurofeedback training following a previously detailed protocol (Hampson et al., 2012a). Of the five OCD patients, the first two underwent only a single neurofeedback session, without pre- or post-neurofeedback resting-state scans. All other individuals participated in four separate MRI scanning sessions, spaced several days apart. In the first session, rs-fMRI data were collected and a functional localizer was used to identify the target area of the OFC/BA 10 region to be used for neurofeedback. The second and third sessions involved rt-fMRI neurofeedback training based on the target OFC/BA 10 region. A final session (not of relevance to this work) involved collecting post-intervention rs-fMRI data. The rs-fMRI data were always collected before any other functional scans in a given session to avoid possible effects of previous task on the rs-fMRI data.

**Figure 1:**
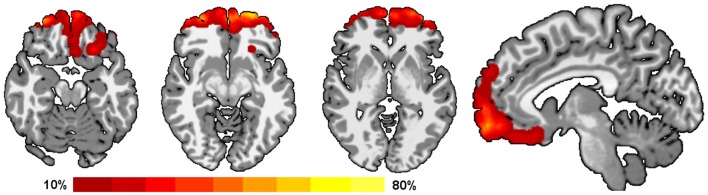
**Overlap of target regions for neurofeedback.** All subjects received neurofeedback from a region in the OFC/BA 10 (Hampson et al., 2012a). These target regions were determined on individual basis from a functional localizer task allowing for differences in individual functional anatomy. The percent overlap of all these target regions is shown on a template brain using Radiological convention (left is on the right for axial slices). Warmer colors indicate that the voxel was included in a greater number of individual target regions.

The overlap of the target area for feedback for all 15 subjects is shown in Figure 1. Overlap was calculated by (1) smoothing the target region of each individual with a 6 mm Gaussian smoothing kernel to account for differences in functional anatomy and registration errors; (2) warping the target regions to a common reference; and (3) averaging across subjects the likelihood of a voxel being included in the target region.

### Behavioral measures

Behavioral measures of control over contamination anxiety (for the first cohort) and clinical measures (for the second cohort) were collected before and after neurofeedback training. The pre-intervention assessment data was collected before the first neurofeedback session (second overall imaging session), immediately prior to the start of neurofeedback training. The post-intervention assessment data was collected several days after the completion of neurofeedback training, either in a separate session with no imaging (for the first two OCD patients) or in conjunction with the fourth fMRI session (for all other patients). Finally, midpoint assessment data was collected in between the first and second neurofeedback sessions for the subjects who received two sessions of neurofeedback.

For the healthy subjects, with subclinical contamination anxiety we used a behavioral measure designed to assess the subjects’ ability to control their anxiety. Subjects were instructed to try to control their anxiety while viewing 25 contamination-related images and to indicate their experienced anxiety for each image on a 1–5 scale. A rating of one indicated the least anxiety and a rating of five indicated the most anxiety. The ratings for the 25 contamination-related images were then averaged yielding a single measure of anxiety. Different sets of images were used before and after the intervention, but the sets were designed to induce similar levels of contamination related anxiety and piloted to verify that they were balanced in this respect (Hampson et al., 2012a).

For the patients, we administered a modified version of the Yale–Brown Obsessive Compulsive Scale (Y-BOCS), in which they were instructed to report on their symptoms over the last 3 days, rather than over the past week as in the traditional Y-BOCS (Goodman et al., 1989a,b). The Y-BOCS ranges from 0–40, with higher scores representing more severe symptoms, and measures the frequency, intrusiveness, and distress associated with obsessions and compulsions. Scores in the mid-twenties, as these patients had (Table 1), correspond to moderate to severe disease.

For both groups, change in behavior measures were calculated as score prior to neurofeedback minus score after neurofeedback, such that a positive change indicates an improvement in anxiety.

### Imaging parameters

All imaging was done on a 1.5-T Siemens Sonata scanner (Siemens Medical Systems, Erlangen, Germany). A sequence designed to optimize signal in the OFC was used for all functional data collection (repetition time = 2000 ms, echo time = 30 ms, flip angle = 80, bandwidth = 2604, 200 mm field of view for 3.1 mm isotropic voxels, 31 axial-oblique slices covering almost the whole cerebrum and most of the cerebellum). Two 5 min resting data runs were collected.

### Resting-state connectivity

Images were preprocessed using a previously detailed pipeline (Hampson et al., 2012b). All images were slice time and motion corrected using SPM.^1^ Unless otherwise specified, all further analysis was performed using BioImage Suite (Joshi et al., 2011). Several covariates of no interest were regressed from the data including linear and quadratic drift, six rigid-body motion parameters, mean cerebrospinal fluid (CSF) signal, mean white-matter signal, and mean global signal. The data were low-pass filtered via temporal smoothing with a 0 mean unit variance Gaussian filter (approximate cutoff frequency = 0.12 Hz). Finally, a gray matter mask was applied to the preprocessed data so that only voxels in the gray matter were used in subsequent calculations. After preprocessing, all resting-state runs were concatenated and the connectivity for each voxel was then calculated in each subject’s individual brain space.

The gray and white matter and CSF masks were defined on a template brain (Holmes et al., 1998), and warped to individual subject space using a series of transformations, described below. The gray matter mask was dilated to ensure full coverage of the gray matter after warping into individual subject space. Regions that were not included in all subjects’ data (for e.g., the bottom of the cerebellum) were excluded from analysis. Likewise, the white matter and CSF masks were eroded to ensure only pure white matter or CSF signal were regressed from the data.

Global functional connectivity of each voxel was measured from rs-fMRI data using the network theory measure *degree* (Bullmore and Sporns, 2009) as previously described (Martuzzi et al., 2011). The BOLD time course for each voxel was correlated with every other voxel in the gray matter. Two voxels were considered connected if correlation of their timecourses was greater than *r* = 0.25; the* degree* of each voxel was defined as the number of such connections. The process was repeated for every voxel in the gray matter. Each subject’s degree map was normalized by subtracting the mean across all voxels and dividing by the standard deviation across all voxels. This normalization has been shown to reduce the impact of confounds related to motion (Yan et al., 2013).

To facilitate comparisons of imaging data, all degree maps were spatially smoothed with a 6 mm Gaussian filter and then warped to a common template space through the concatenation of a series of linear and non-linear registrations, as previously described (Scheinost et al., 2013). All transformations were computed using the intensity-based registration algorithms in BioImage Suite (Papademetris et al., 2004).

### Evaluating the relationship between response to intervention and rs-fMRI data

To identify which brain regions predicted response to neurofeedback training, we related the rs-fMRI data acquired before any neurofeedback training with changes in the behavioral measure of control over contamination anxiety (for the healthy subjects) and changes in clinical severity (for the patients). For the healthy subjects, we performed a data-driven, whole-brain analysis by correlating the change in control of anxiety with the *degree* maps in a voxel-wise manner. Significance was assessed at a *p* < 0.05 level after correcting for multiple comparisons across the gray matter via AFNI’s AlphaSim program. From this voxel-wise analysis, we defined a region of interest (ROI) that showed significant effects in the healthy subjects to explore whether this finding translated to the smaller cohort of OCD patients. For the three OCD patients on whom pre-neurofeedback rs-fMRI was collected, *degree* averaged over all voxels in this ROI was extracted and related to changes in Y-BOCS scores.

## Results

### Imaging predictors of behavior in subclinically anxious subjects

**Figure 2:**
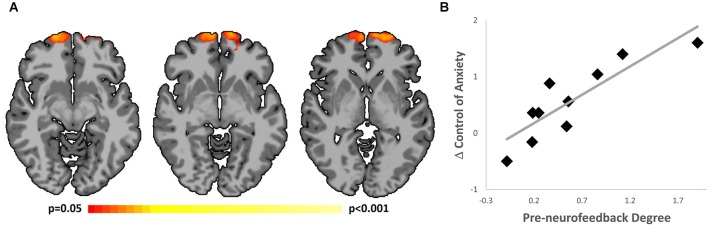
**Correlation of improved control over anxiety and rs-fMRI. (A)** Subjects with the highest connectivity in the OFC/BA 10 (MNI coordinate of peak voxel: 0, 66, −4) prior to neurofeedback training had the largest improvement in control over anxiety over the course of the intervention. Results shown using Radiological convention at *p* < 0.05 level, corrected for multiple comparisons. **(B)** Scatterplot showing improved control over anxiety and pre-neurofeedback rs-fMRI.

As reported previously (Scheinost et al., 2013), healthy subjects with subclinical contamination anxiety showed a significant (*p* < 0.05) increase in control over anxiety after neurofeedback training. Whole-brain connectivity analysis revealed a single significant cluster (*p* < 0.05 corrected; MNI coordinate of peak voxel: 0, 66, −4, max *t*-value = 5.84, cluster size = 5857 mm^3^) in which *degree* prior to neurofeedback training was significantly correlated with improved control over anxiety (Figure 2A). This cluster was located in the OFC/BA 10 target region. Subjects with the highest connectivity in this region prior to neurofeedback training exhibited the most improvement in post-treatment anxiety. A scatterplot of the average connectivity change in this region vs. the change in control of anxiety is shown in Figure 2B. As the choice of threshold used to consider whether two voxels are connected can impact connectivity results (Scheinost et al., 2012), we repeated this analysis over a range of thresholds (0.10 < *r* < 0.65). This produced no qualitative change in the findings. Additionally, as motion has been shown to confound functional connectivity results, average frame to frame displacement was calculated for each group (Van Dijk et al., 2012). Motion was not correlated with improved control of anxiety (*r* = 0.18, *p* > 0.60) and adding motion as a covariate in the group analysis did not change the presented results.

### Clinical improvement after neurofeedback in subjects with OCD

Five patients with moderate-to-severe OCD and prominent contamination symptoms underwent one or two sessions of neurofeedback (Table 1). All five tolerated the procedure well and exhibited reduced symptoms, as evaluated by the Y-BOCS several days after the last neurofeedback session. Average symptom improvement was 20%. Of the OCD patients, the three with the greatest symptom improvements also had a co-diagnosis or a history of major depressive disorder (MDD). Demographic and clinical details are given in Table 1.

### Imaging predictors of clinical improvement

Next, we tested whether a similar relationship between connectivity and behavioral improvements would be found in OCD patients. Pre-neurofeedback rs-fMRI was not measured on the first two subjects; this analysis was therefore performed only on the three subjects who underwent the full two-session neurofeedback protocol. To maximize power in this very limited dataset, we used the OFC/BA 10 region defined in the first cohort as an *a priori* ROI, Average *degree* in this ROI prior to neurofeedback training was related to clinical improvement for the three patients. Consistent with the pattern seen in the healthy subjects, a strong linear relationship was observed (*r* = 0.99). Thus, in both groups, increased connectivity in the OFC/BA 10 measured from rs-fMRI data collected prior to neurofeedback training was associated with greater behavioral improvements.

## Discussion

Advances in understanding individual differences motivate a new approach to health care in which treatment is tailored to the specific needs and biology of an individual patient. This “personalized medicine” approach has been endorsed by the National Institute of Mental Health, NIMH,^2^ but its adoption depends critically on our ability to identify which patients are likely to respond to which interventions. rs-fMRI holds great promise as a tool for providing this information. It is easy to collect, does not require patients to perform any difficult tasks, and yet is a rich source of potentially clinically relevant information about brain function (Constable et al., 2013; Lee et al., 2013).

In a pilot study, we demonstrate, for the first time, that rs-fMRI can be a useful tool to predict response to neurofeedback training via rt-fMRI. After receiving two sessions of neurofeedback training, healthy subjects showed improved control over anxiety and OCD patients showed a reduction in OCD symptom severity. For both groups, these behavioral improvements were strongly correlated with the pre-intervention level of whole-brain connectivity in the anterior prefrontal cortex.

The resting state functional connectivity analysis used in this study was unbiased by *a priori* expectations regarding regions of interest. Therefore, it is striking that the region that emerged from our whole-brain analysis as most relevant for predicting improvements in contamination anxiety was in our target area of the OFC/BA 10. Taken together with a large body of data highlighting the importance of the OFC and anterior prefrontal cortex in obsessive-compulsive symptoms (Swedo et al., 1992; Chamberlain et al., 2008; Menzies et al., 2008; Harrison et al., 2009; Sakai et al., 2011; Anticevic et al., 2014; Beucke et al., 2013), this gives us confidence that we are targeting a biologically relevant brain area.

Notably, OFC/BA 10 connectivity predicted the response to the intervention in both healthy subjects and OCD patients, suggesting a shared neurobiological mechanism for improved control over contamination anxiety across groups. It is possible that the phenomenon of contamination anxiety is a dimensional construct, differing in a quantitative rather than a qualitative sense in patients when compared to healthy individuals. Supporting this view are previous reports of OFC/BA 10 activations to contamination related imagery in both healthy subjects and OCD patients (Mataix-Cols et al., 2003, 2004).

To the extent that neurobiology of a phenomenon is shared across patients and healthy subjects, interventions developed in the healthy group are likely to translate into the patient population. In this particular intervention, based on our preliminary patient data, translational potential appears high. A variety of other applications of rt-fMRI neurofeedback trainings have been developed in healthy populations (Hampson et al., 2011; Shibata et al., 2011; Chiew et al., 2012; Garrison et al., 2013). It will be interesting to see how well the findings from these studies translate into clinical populations. If the dimensional approach implicit in NIMHs Research Domain Criteria^3^ is an accurate description of pathological brain dysfunction, many of these studies may successfully translate into the respective patient groups.

An important consideration for predictive validity is the reliability of rs-fMRI. Overall, graph theory measures have been shown to be reliable (Telesford et al., 2010; Braun et al., 2012) and, in particular, voxel-wise degree has shown good test-retest reproducibility across different sites and scanners (Tomasi and Volkow, 2010). While generally reliable, a variety of factors can reduce the predictive power of rs-fMRI. Medications and other drugs such as caffeine can alter connectivity patterns (Rack-Gomer et al., 2009; Martuzzi et al., 2010). Sleep also changes connectivity patterns (Tagliazucchi et al., 2013) which can be an issue if subjects are falling asleep and not reporting it. Finally, factors related to subject comfort such as hunger may reduce data quality and prediction accuracy due to motion artifacts and effects on subject compliance. The degree to which all these variables are controlled is likely to affect the power of future studies to identify clinically relevant biomarkers that predict treatment response.

The major limitation of this pilot study is the small number of subjects, particularly in the patient group, in which we only had three subjects with resting data. Although the finding in the healthy subject group is statistically significant, the finding in the patient group must be considered preliminary. However, the tight correspondence between connectivity and intervention response in our modest clinical sample, and its similarity to the relationship seen in healthy subjects, are promising. Future studies are needed to rigorously examine whether this biomarker is an effective predictor of response in the clinical group. A large study that can examine possible modulating variables would be particularly valuable. For example, the data in our small sample suggest that patients with a current co-diagnosis or a history of MDD show the greatest improvement in clinical symptoms, but we were unable to investigate this given our limited data in the patient group. A study with the power to test that possibility could yield interesting insights.

## Conclusion

These pilot data provide evidence that rs-fMRI connectivity can be used to identify individuals likely to benefit from rt-fMRI neurofeedback interventions for training control over contamination anxiety. Specifically, we have identified a biomarker that may be useful in developing personalized treatment programs in patients with OCD. More generally, these findings illustrate the potential utility of rs-fMRI data for identifying biomarkers of treatment response and thereby facilitating a personalized medicine approach to treating mental illness.

## Conflict of interest statement

The authors declare that the research was conducted in the absence of any commercial or financial relationships that could be construed as a potential conflict of interest.

## Footnotes

^1^http://www.fil.ion.ucl.ac.uk/spm/

^2^http://www.nimh.nih.gov/about/strategic-planning-reports/index.shtml

^3^http://www.nimh.nih.gov/research-priorities/rdoc/nimh-research-domain-criteria-rdoc.shtml
